# Supplementary material for: Molecular Imaging of Oxygenation Changes during Immunotherapy in Combination with Paclitaxel in Triple Negative Breast Cancer
Source: Biomedicines. 2023 Jan 4;11(1):125. doi: 10.3390/biomedicines11010125 (PMC9856084; doi:10.3390/biomedicines11010125)
Supplement: Supplementary file 1 [file biomedicines-11-00125-s001.zip › biomedicines-2073176-supplementary.pdf]

SUPPLEMENT

Expanded Methods: Flow Cytometry

Following compensation, forward scatter height (FSC-H) on the x-axis versus side scatter height (SSC-H) was the gating strategy used to identify all cells. Next, FSC-H × FSC-area (A) was used to gate for single cells, SSC-H × SSC-A was used to gate for singlets, Dead × SSC-A was used to gate for live cells/dead negative, and CD45 × SSC-A was used to gate for CD45+ populations of hematopoietic derived cells (lymphocytes). F4/80 × SSC-A was used to gate for all macrophages. CD80 and CD206 × SSC-A were used to gate for M1 and M2 macrophages, respectively. CD3 × SSC-A was used to gate for all T-cells. CD4 and CD8 × SSC-A were used to gate for CD4+ and CD8+ T-cells, respectively. CD4 × CD25 was used to gate for proliferating CD4+ T-cells.

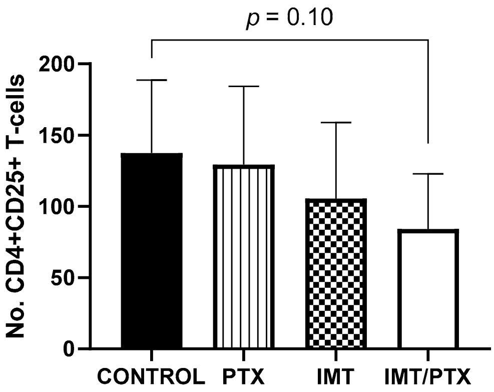

Supplemental Figure S1. No significant differences in proliferating CD4+ T-cells were found between treatment groups. Treatment of TNBC tumors with combination IMT/PTX decreased the numbers of proliferating CD4+ T-cells, indicated by CD25+ positivity, compared to untreated control tumors; however, no significant differences in proliferating CD4+ T-cell numbers were observed between treatment groups. Mean ± SD (multiple t-tests).
